# The American Medical Association

**Published:** 1889-08

**Authors:** 


BUFFALO MEDICAL AND SURGICAL JOURNAL

A MONTHLY REVIEW OF MEDICINE AND SURGERY.

EDITORS:

THOS. LOTHROP, M. D. - - - W. W. POTTER, M. D.

All communications, whether of a literary or business character, should be addressed to the editors: 284 FRANKLIN STREET, BUFFALO, N. Y.

### Editorial.

### *THE AMERICAN MEDICAL ASSOCIATION.*

WHEN we wrote last month of the Newport meeting of this Association, we were of the opinion that nothing could grow out of that meeting to mar its delightful memory.

But the following extract from the letter of the special correspondent, "P. B. P.," of the *Journal of the American Medical Association*, published in its issue of July 20th, demands some comment, namely:

"The election of Dr. E. M. Moore, of Rochester, as President of the American Medical Association, meets with much enthusiasm here, and, aside from the fact that nowhere in the profession could there be found one more eminently qualified by natural gifts and special culture to fill the position with dignity and grace, *it is felt that the selection is in some sense a recognition of the loyalty of that portion of the New York profession which has always stood true to the National colors*, and of which Professor Moore is one of the most distinguished ornaments."

We have italicised that portion of the paragraph to which we take exception.

It would appear, it seems to us, in the minds of all right-thinking persons, that it is high time that the childish prattle of this "P. B. P." should be suppressed. We are now at a period in the history of American medicine when it is about time to drop all politico-medical jugglery, and settle down upon the broad platform of science. One of the reasons why the Newport meeting was one of the best, if not the very best ever held by the American Medical Association, is that it divested itself for the nonce of every appearance of vindictiveness, and there were certainly no acrimonious debates upon questions foreign to the real purposes of the meeting. Those questions which none but the smaller minds in the high councils of the Association care one whit about, did not come to the surface for discussion.

The vast majority of the men who were present at Newport were intent only upon doing good work, caring very little who wielded the gavels in the several meetings, or what the Judicial Council considered in closed session. The men present from New York were from both State medical organizations, working in harmony for the general good, and particularly for the honor of American medicine. It is true that Dr. Moore has been an officer in the New York State Medical Association, and it is also true that the men of that faction supported him with earnestness; but it is equally true that the men of the Medical Society of the State of New York, who were present, worked with just as creditable zeal to the end that Dr. Moore might occupy a position which his learning and talents entitled him to, and which he would long ago have held had it not been for the indiscretion of just such men as "P. B. P."

We yield to no man in our admiration for Dr. Moore. We have said that he is an ideal surgeon, and we go further and say that we believe him to be an ideal man. He is the one man of all others in this broad domain, where great men in medicine are plentiful, to whom our eyes and thoughts turn in admiration and thankfulness that he has been elected to this high and honorable office; but at this particular juncture of affairs, when everything is tending to peace and harmony, when there are really no divisions in the State of New York on questions of a scientific nature, except such as pertain to honorable differences always among men, we repeat that it is in the very worst kind of taste for the *Association Journal* to retail the puerile utterances of its paid correspondent, "P. B. P.," who presumes to speak by the card for everything that is "good and loyal" amongst the medical profession of the State of New York.

The senseless editorial on page 164 of the *Journal* for February 2, 1889, was allowed to pass in comparative silence, as far as the medical press of the State of New York was concerned, and after its stinging rebuke by the editor of *Progress*, we did think that we should see no more writing of that sort in its columns. We happen to know that the trustees of the *Journal*, or at least some of them, disapproved of that editorial, and we hope that, at least while they are assuming its editorial control, they will see to it that "P. B. P." and all of his kidney that have to do therewith in any way, are duly suppressed.

---

THE *St. Louis Weekly Medical Review* begins Volume XX. with its issue of July 6, 1889, in an enlarged form and with newly-arranged pages. In its present size it resembles the quarto weeklies of the eastern cities, while its subscription price is maintained at $3.50 a year.

